# Identification of the Key Pathways and Genes in Hypoxia Pulmonary Arterial Hypertension Following Intrauterine Growth Retardation

**DOI:** 10.3389/fmolb.2022.789736

**Published:** 2022-03-31

**Authors:** Weifen Zhu, Ziming Zhang, Weiwei Gui, Zheng Shen, Yixin Chen, Xueyao Yin, Li Liang, Lin Li

**Affiliations:** ^1^ Department of Endocrinology, The Affiliated Sir Run Run Shaw Hospital, School of Medicine, Zhejiang University, Hangzhou, China; ^2^ Department of Neonatology, Children’s Hospital of Zhejiang University School of Medicine, Hangzhou, China; ^3^ Department of Central Laboratory, Children’s Hospital of Zhejiang University School of Medicine, Hangzhou, China; ^4^ Department of Pediatrics, The First Affiliated Hospital, School of Medicine, Zhejiang University, Hangzhou, China

**Keywords:** pulmonary arterial hypertension, intrauterine growth retardation, hypoxia, weighted gene co-expression network analysis, metabolic dysfunction

## Abstract

High-throughput sequencing and weighted gene co-expression network analysis (WGCNA) were used to identify susceptibility modules and genes in liver tissue for the hypoxic pulmonary arterial hypertension (PAH) animal model following intrauterine growth retardation (IUGR). A total of 5,000 genes were clustered into eight co-expression modules *via* WGCNA. Module blue was mostly significantly correlated with the IUGR–hypoxia group. Gene Ontology analysis showed that genes in the module blue were mainly enriched in the fatty acid metabolic process, lipid modification, and fatty acid catabolic process. The Kyoto Encyclopedia of Genes and Genomes enrichment analyses showed that the genes in module blue were mainly associated with fatty acid metabolism, PPAR signaling pathway, and biosynthesis of unsaturated fatty acids. In addition, the maximal clique centrality method was used to identify the hub genes in the subnetworks, and the obtained results were verified using real-time quantitative PCR. Finally, we identified that four genes including Cyp2f4, Lipc, Acadl, and Hacl1 were significantly associated with IUGR-hypoxia. Our study identified a module and several key genes that acted as essential components in the etiology of the long-term metabolic consequences in hypoxia PAH following IUGR.

## 1 Introduction

Pulmonary arterial hypertension (PAH) is a life-threatening disease characterized by increased vascular resistance and pressure, which ultimately results in right heart failure (Schermuly et al., 2011). With the deepening of research, PAH is increasingly being recognized as a systemic illness that can lead to the metabolic derangements, such as insulin resistance, hyperglycemia, and dyslipidemia (Zamanian et al., 2009; Heresi et al., 2010; Pugh et al., 2011; Assad and Hemnes 2015). Nowadays, some studies have provided strong evidence that PAH might have its origins in fetal or early life as a result of an adverse intrauterine environment during sensitive periods of development (Rexhaj et al., 2011; Napoli et al., 2019). Our previous study also discovered that intrauterine growth retardation (IUGR) as a result of undernutrition is related to hypoxia PAH (Xu et al., 2013). As is known to all, IUGR is highly correlated with metabolic diseases. Therefore, we speculate that there will be significant changes in metabolomic characteristics in hypoxia PAH following IUGR. Based on this, we stared this research with special attention to this point.

Transcriptome-wide profiling has enabled comprehensive study of the entire transcriptome of mRNAs, microRNAs, and long non-coding RNAs, which has the potential to uncover the complex cellular phenotypes, biological processes, and metabolomic characteristics (Chen et al., 2020). In addition, the identification of differentially expressed genes also contributes to uncovering crucial genes associated with disease. Weighted correlation network analysis (WGCNA), as one of the most widely used methods of systems bioinformatics that explores modular information, can be utilized for constructing weighted correlation networks and finding clusters of highly correlated genes (Langfelder and Horvath 2008; Wang et al., 2019; Yang and Li 2019). Several microarray-based gene expression studies have been conducted on PAH in recent years (Mura et al., 2012; Luo et al., 2020). However, most analytical results focused on the pathogenesis of PAH, and not the metabolic characteristics. Thus, we aimed to perform WGCNA based on data obtained from RNA-seq data to identify critical co-expression modules and pathways correlated with clinical characteristics of hypoxia PAH following IUGR in the liver. The results obtained in this study might provide new insights into the metabolic characteristics of hypoxia PAH following intrauterine environmental changes.

## 2 Methods

### 2.1 Intrauterine Growth Retardation and Hypoxia Pulmonary Hypertension Rat Model

**FIGURE 1:**
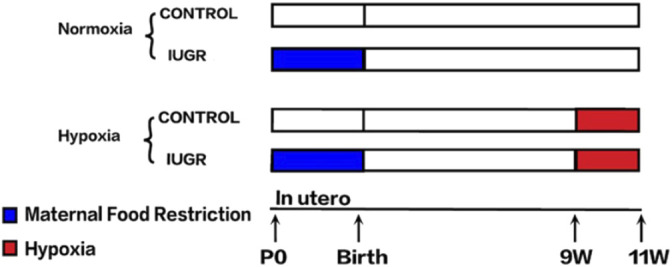
Flowchart of the experimental procedure.

The animal model used in this study had been successfully constructed in our previous study, the flowchart of the experimental procedure is displayed in Figure 1 (Zhang et al., 2019). Briefly, all animal procedures were performed in accordance with the guidelines of the Animal Ethics Committee of Zhejiang University. Timed mating was performed in virgin Sprague–Dawley females weighing 250 ± 10 g (mean ± SD) obtained from Slack Experimental Animal Center of Chinese Academy of Sciences (Shanghai, China). After confirmation of pregnancy by vaginal smear plug, the females were randomly divided into two groups with an *ad libitum* diet of standard laboratory chow or a 50% food-restricted diet determined by the quantification of normal intake in *ad libitum*-fed rats from day 1 of pregnancy until parturition. The offspring delivered by rats with normal food intake were defined as the control group, while the offspring delivered by food-restricted rats with a birth weight below the 10th percentile of controls were defined as IUGR. To avoid hormonal cycle disturbances, only male pups were further investigated. All pups were then weaned onto standard chow at 3 weeks. At 9 weeks of age, the IUGR and control offspring were randomly divided into two groups. The normoxia group was placed in a normal room air chamber, while the hypoxia group was placed in the hypoxia chamber with the fraction of inspired oxygen of 11% for 2 weeks. These subdivisions yielded two subgroups per group: control-normoxia (*n* = 8), control-hypoxia (*n* = 8), IUGR-normoxia (*n* = 8), and IUGR-hypoxia (*n* = 8). Rats in all the groups were fed with standard chow *ad libitum* and were housed in separate cages maintained with constant temperature and humidity. The cages and soda lime in the chambers were changed daily to prevent the accumulation of excess ammonia and CO_2_, respectively. Samples from the liver were harvested and snap-frozen in liquid nitrogen, and five samples were selected randomly each from control-normoxia, control-hypoxia, IUGR-normoxia, and IUGR-hypoxia for subsequent processing.

### 2.2 RNA Extraction, Library Construction, and Transcriptome Sequencing

Total RNA was isolated from the liver using TRIzol reagent (Invitrogen Life Technologies), followed by the determination of the quality and integrity using a NanoDrop spectrophotometer. First-strand cDNA was synthesized using random hexamers and SuperScript II, while second-strand cDNA synthesis was subsequently performed using DNA polymerase I and RNase H. The second-strand cDNA was then purified and treated using terminal repair and ligation primers. Products were purified (AMPure XP beads) and quantified using the Agilent high-sensitivity DNA assay on a Bioanalyzer 2100 system (Agilent). After selection using AMPure XP beads, the second-strand cDNA containing U was degraded by the USER enzyme. PCR amplification was then performed, followed by sequencing of the library using Illumina HiSeq sequencing by Whbioacme Co. Ltd.

### 2.3 Weighted Co-Expression Network Construction and Module Division

First, we excluded the samples that emerged as clear outliers using the hierarchical clustering method. Second, we applied WGCNA with the expression profile using the R package of “WGCNA” (Langfelder and Horvath, 2008). Pearson’s correlations were performed for all pair-wise genes, followed by the construction of a correlation matrix. The correlation matrix was then transformed into an adjacency matrix (also known as the scale-free network) using an appropriated soft thresholding powers *β*. The *β* value was set from 1 to 20, followed by the calculation of the scale-free fit index and mean connectivity for each *β* value. The *β* value was determined when the scale-free fit index was up to 0.85, and the *β* value that had the highest mean connectivity was considered to be the most appropriated one. The topological overlap matrix (TOM) was then obtained on the basis of the adjacency matrix. Finally, the average linkage hierarchical clustering was conducted according to the TOM-based dissimilarity measure in order to divide all genes into several co-expression modules having gene module sizes above 30. We then calculated Pearson’s correlations of the eigengenes after defining the first principal component of a given module to further analyze the module. The modules whose eigengenes were highly correlated were merged into one module, with Pearson’s correlation being higher than 0.75.

### 2.4 Identification of Clinically Meaningful Modules

The clinical traits of our samples included IUGR and hypoxia. The correlation between modules and traits was calculated, and the modules having a positive correlation with IUGR and hypoxia were considered as playing roles in the pathogenesis of the disease. On the other hand, genes in modules having a positive correlation with a normal trait were indispensable for maintaining normal biological functions. Thus, we extracted gene modules having the highest correlation with IUGR and hypoxia for subsequent studies.

Gene significance (GS) and module membership (MM) were introduced to the correlation of a given gene with a clinical trait and module eigengene, respectively. Genes in clinical-related modules should have high values and preferable correlations of GS and MM.

### 2.5 Functional Enrichment Analysis

We used clusterProfiler R package (version 3.15.3) to perform Gene Ontology (GO) enrichment analysis and Kyoto Encyclopedia of Genes and Genomes (KEGG) pathway enrichment analysis in order to explore the biological functions of genes in the clinical related modules (Shannon et al., 2003). The *p*-value < 0.01 and Benjamini–Hochberg adjusted *p*-value (Benjamini and Hochberg 1995) <0.01 were considered to be statistically significant.

### 2.6 Identification of Candidate Hub Gene

Generally, the hub genes in the co-expression network should have high connectivity with the whole module and clinical trait. For identifying the hub gene related with IUGR-hypoxia, we extracted gene clusters that were enriched in certain GO terms from the WGCNA network to construct subnetworks after GO enrichment analysis of the IUGR-hypoxia-related module. The CytoHubba Cytoscape plugin was then used to identify hub genes (Chin et al., 2014). This study regarded the genes with high Maximal Clique Centrality (MCC) values as being hub genes because MCC has a better performance in predicting hub genes.

### 2.7 Validation of the Hub Genes Using qRT-PCR

We validated the expression of hub genes using qRT-PCR from liver tissue in order to obtain further evidence for the significance of hub genes in the module of interest.

The dissected liver was homogenized, followed by the isolation of RNA using TRIzol reagent (Invitrogen) in accordance with the manufacturer’s instructions. The obtained total RNA was then converted to cDNA using a reverse transcription reagent kit (TaKaRa). Real-time quantitative PCR was performed using a 7500 Real-Time PCR Detection System (Applied Biosystems) using a PCR kit containing SYBR Green (TaKaRa). The obtained results were expressed in relative expression using the comparative 2^−∆∆ Ct^ method normalized by the housekeeping gene GAPDH. Statistical significance was calculated using Student’s *t*-test by SPSS 18.0 (SPSS, Inc., Chicago, IL, United States). *p* < 0.05 was considered to indicate a statistical difference (Table 1).

**Table 1:** The primer sequences of the hub genes.

Gene	Sequence	Product
MAPK14	Forward	GCTGGCTCGGCACACTGATG
	Reverse	GCCCACGGACCAAATATCCACTG
Fam126b	Forward	GAGCCTGTCTGCCACCAACTG
	Reverse	GCCATTGCTCTGCCTGTCTCTAC
Cyp2j10	Forward	CGCTGCTGTCACCTTCCTGTTC
	Reverse	TGGCTGCTTCACATCCAACTGG
Hacl1	Forward	CATGTTCGGTGTCGTAGGCATCC
	Reverse	GCCGCTTGCTCATTCCTCATCC
Acsm3	Forward	CTGTCTGTCAACGGAAGGTTCTGG
	Reverse	AAACACATGCTCCTTGGGTCCAC
Cyp2j4	Forward	AGAGCTTGCCTTGGAGAACAACTG
	Reverse	GCGGTGCGTGACTGGAGAAAG
STARD4	Forward	CCTGCGGCTGGTTCTGTGTTC
	Reverse	TGCTTGCCATTGCTGTGTCTACC
ACSL5	Forward	GGCATCATTCGGCGGAACAG
	Reverse	TGCAGCCCTGAAGAACGTCA
ACADVL	Forward	TGTGCTAGGAGAAGTGGGAGATGG
	Reverse	TCAACCGCCTTGGCAATGATGG
AGTR1a	Forward	GCTTCAACCTCTACGCCAGTGTG
	Reverse	CGAGACTTCATTGGGTGGACGATG
Cyp2f4	Forward	TGTCATCTTCGGCAGTCGTTTCG
	Reverse	CCAGGCACCCAGTCCAGGAG
Acads	Forward	CTCACAGCAGAAGCAGCAGTGG
	Reverse	TGCCGTTGAGGACCCAGGAG
Lipc	Forward	AGGTGGCTGCTCTTCTCCTATGG
	Reverse	GCTCCCAGGCTGTACCCAATTAAG
ACAT1	Forward	CAGACGTGGTGGTGAAGGAAGATG
	Reverse	ATCGTTCAGTGTGCTGGCGTTAG
Acadl	Forward	CCCTGGTTTCAGCCTCCATTCAG
	Reverse	CACTTGCCCGCCGTCATCTG
HADHA	Forward	GGTGTCTTGCTCCCATGATGTCAG
	Reverse	GAAGCCGAAGCCTGTGGTCAAG
ECI1	Forward	CCGAGCGTGCCCTTCAACTG
	Reverse	GCCATCACTGAGCGAGCCTTG
GAPDH	Forward	GACAACTTTGGCATCGTGGA
	Reverse	ATGCAGGGATGATGTTCTGG

Notes: MAPK14, mitogen-activated protein kinase 14; Fam126b, family with sequence similarity 126 member B; Cyp2j10, cytochrome P450, family 2, subfamily j, polypeptide 10; Hacl1, 2-hydroxyacyl-CoA lyase 1; Acsm3, acyl-CoA synthetase medium-chain family member 3; Cyp2j4, cytochrome P450, family 2, subfamily j, polypeptide 4; STARD4, cytosolic StAR-related lipid transfer domain 4; ACSL5, acyl-CoA synthetase long-chain family member 5; ACADVL, acyl-CoA dehydrogenase, very long chain; AGTR1a, angiotensin type 1a receptors; Cyp2f4, cytochrome P450, family 2, subfamily f, polypeptide 4; Acads, acyl-Coenzyme A dehydrogenase; Lipc, Hepatic lipase; ACAT1, acetyl-coA acetyltransferase 1; Acadl, long chain acyl CoA dehydrogenase; HADHA, hydroxyacyl CoA dehydrogenase trifunctional multienzyme complex subunit alpha; ECI1, Enoyl-CoA Delta Isomerase 1; GAPDH, glyceraldehyde-3-phosphate dehydrogenase.

## 3 Results

### 3.1 Establishment of Hypoxia Pulmonary Arterial Hypertension Rat Model

The mean pulmonary arterial pressure, right ventricular hypertrophy, the media wall area thickness, and pulmonary vascular endothelial function assessment had been shown in our previous study (Zhang et al., 2019), which provided evidence that our model was built successfully.

### 3.2 Data Preprocessing

**FIGURE 2:**
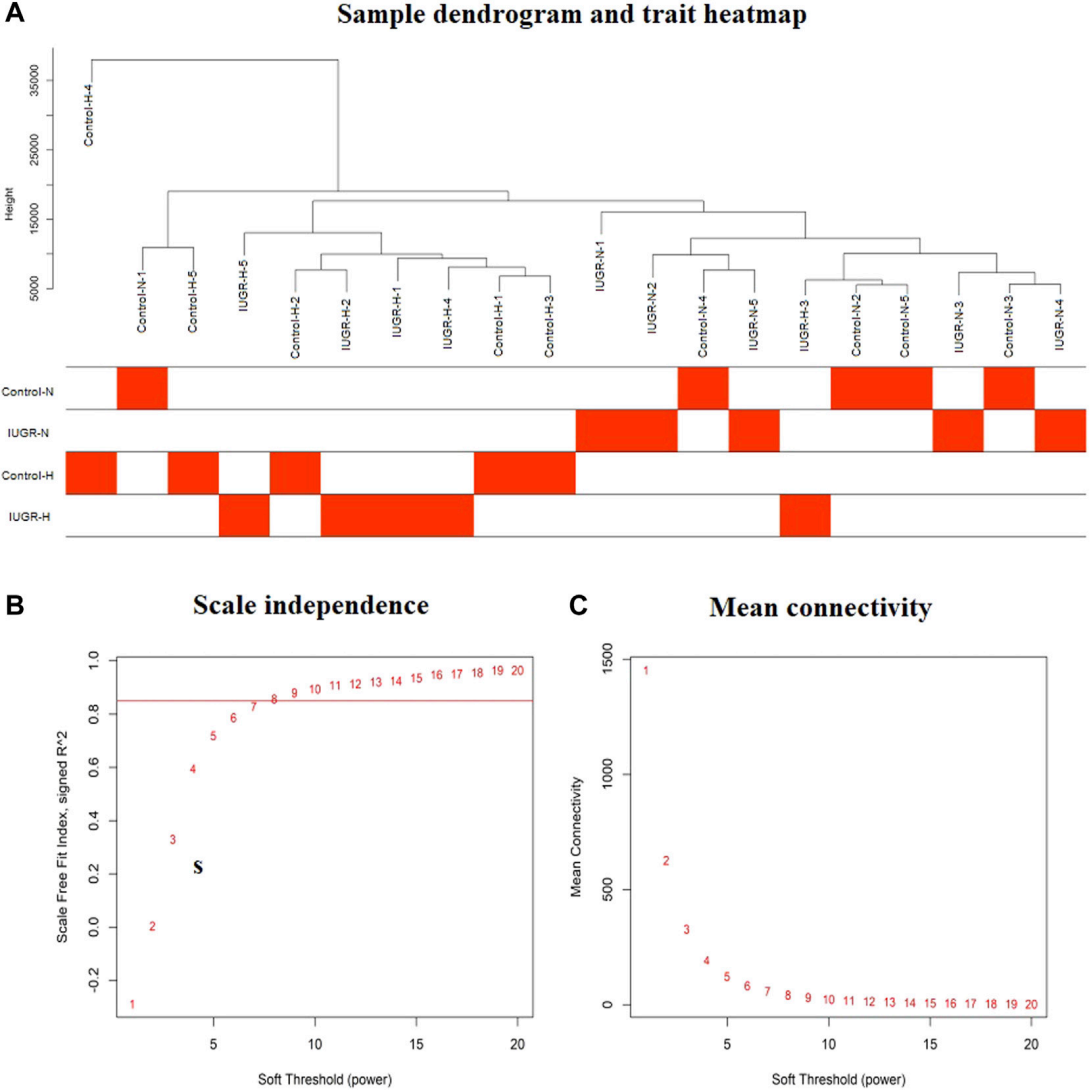
Sample dendrogram and trait heatmap, and the estimation of soft thresholding values (*β*). **(A)** Sample cluster dendrogram and clinical trait heatmap of 20 liver samples based on their expression profile: five control-normoxia (Control-N), five IUGR-normoxia (IUGR-N), five control-hypoxia (Control-H), and five IUGR-hypoxia (IUGR-H). **(B)** Analysis of the scale-free fit index for various soft thresholding powers (*β*). **(C)** Analysis of mean connectivity of various soft thresholding powers.

Since non-varying genes are usually regarded as background noise, in this study, we filtered the genes by variance, and the top 50% (5,000) genes with larger variance were chosen for subsequent analyses. Sample clustering excluded the outlier samples, thereby leaving a total of five control-normoxia samples, five IUGR-normoxia samples, four control-hypoxia, and five IUGR-hypoxia samples. The final results after sample clustering revealed intra-group consistency and intergroup variability, as shown in Figure 2A.

### 3.3 Weighted Co-Expression Network Construction and Module Division

**FIGURE 3:**
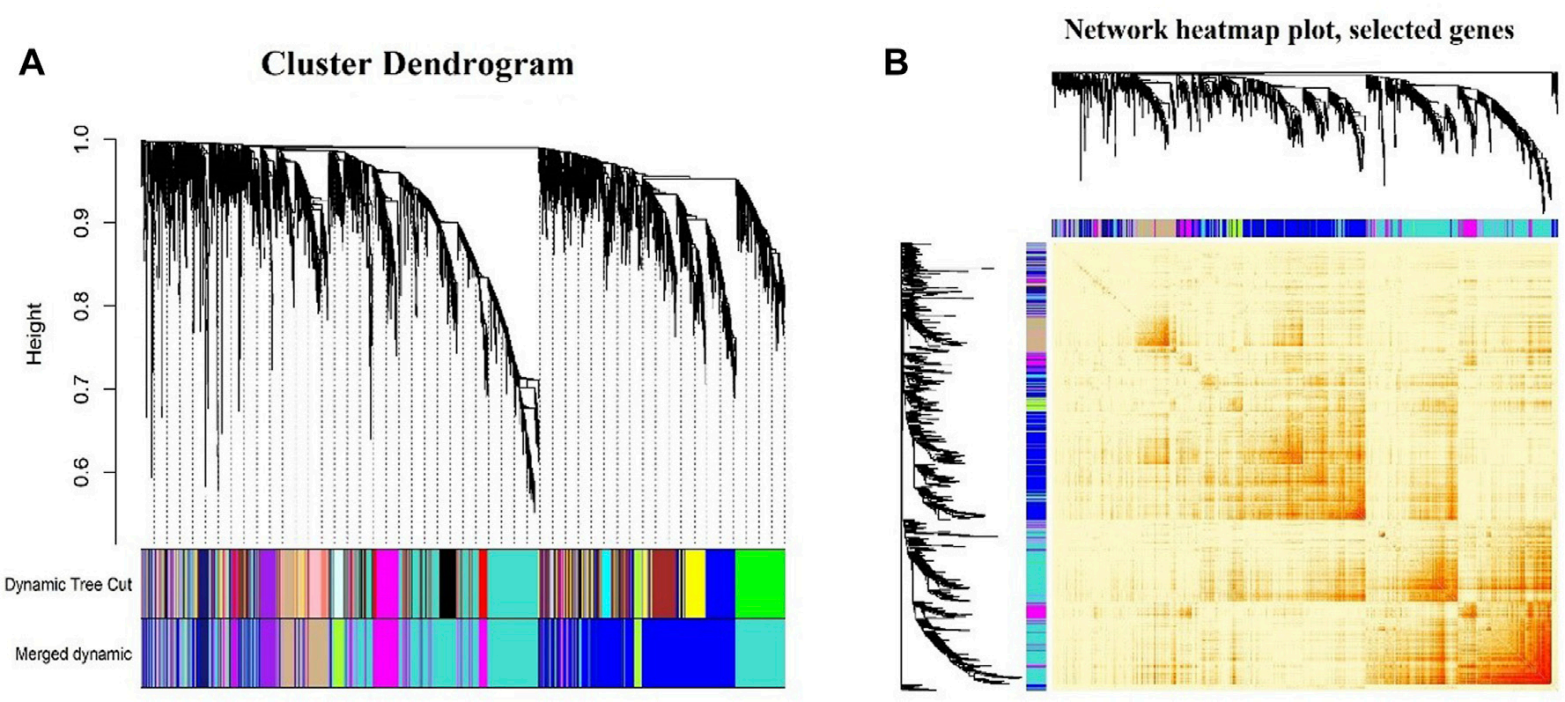
Division and validation of co-expression modules. **(A)** Cluster dendrogram of the identified co-expression modules. The identified modules were coded by colors indicated below the dendrogram. The upper one presents the first set of modules obtained from the dynamic tree cut algorithm, while the lower one presents the merged modules according to Pearson’s correlation analysis. **(B)** Network heatmap plot showing topological overlaps, with light colors denoting low adjacency and darker colors denoting higher adjacency.

In this study, the soft thresholding was determined as eight on the base of the scale-free fit index and mean connectivity (Figures 2B,C). Accordingly, the correlation matrix was transformed to the adjacency matrix and then converted to a topological overlap matrix to improve the sensitivity of co-expression identification. A total of eight co-expression modules with different genes were generated and displayed with different colors based on the average linkage hierarchical clustering and module merging (Figure 3A). The modules included 85 genes in a midnight blue module, 1,641 genes in a blue module, 447 genes in a tan module, 219 genes in a green–yellow module, 179 genes in a purple module, 591 genes in a magenta module, and 1,761 genes in a turquoise module. The gray module contained genes that could not be divided into any co-expression modules.

Figure 3B shows the topological overlap matrix among all genes analyzed by WGCNA; light color represents low overlap, while the progressively darker red color represents a higher overlap.

### 3.4 Identification of Clinically Significant Modules

**FIGURE 4:**
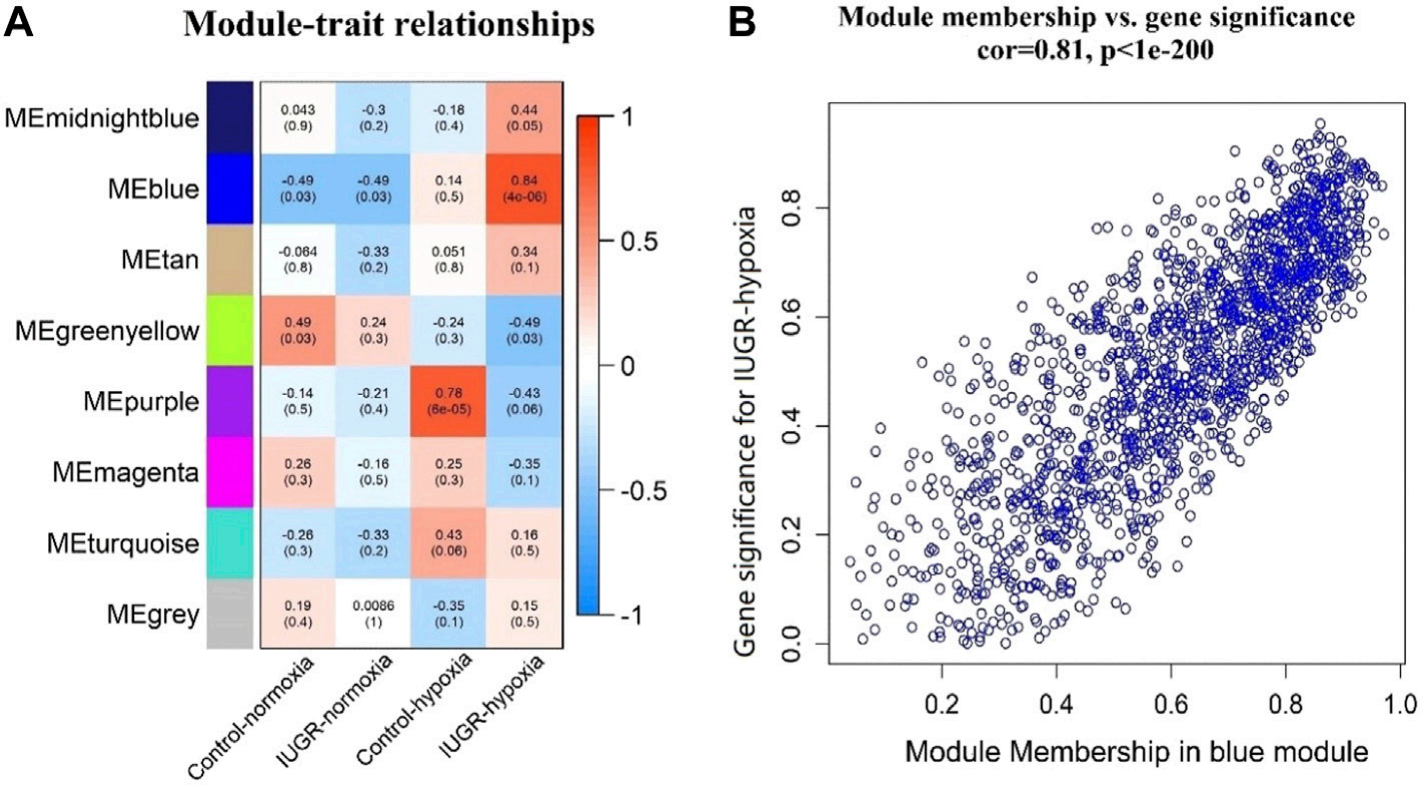
Identification and verification of clinical related modules. **(A)** Module–trait associations. Each row corresponds to a module eigengene, while each column corresponds to a trait. Each cell contained the correlation coefficients and *p*-value. **(B)** Scatterplot of gene significance (GS) vs. module membership (MM) in the blue module. The correlation coefficients and *p*-value are listed above the scatterplot.

The associations between modules and trait were investigated using Pearson’s correlation analysis in the WGCNA, and the obtained results are shown in Figure 4A. The results indicated that the blue module displayed highest correlation with IUGR-hypoxia (*r* = 0.84, *p* = 4e-06). The GS vs. MM values of all member genes of the blue module are shown in the scatterplot (Figure 4B). The GS and MM values were highly correlated in the blue module (cor = 0.81, *p* < 1e-200), indicating that genes in the blue module were highly significantly associated with clinical traits, and thus, they were suitable for further analyses.

### 3.5 Gene Ontology and Kyoto Encyclopedia of Genes and Genomes Enrichment Analyses

**FIGURE 5:**
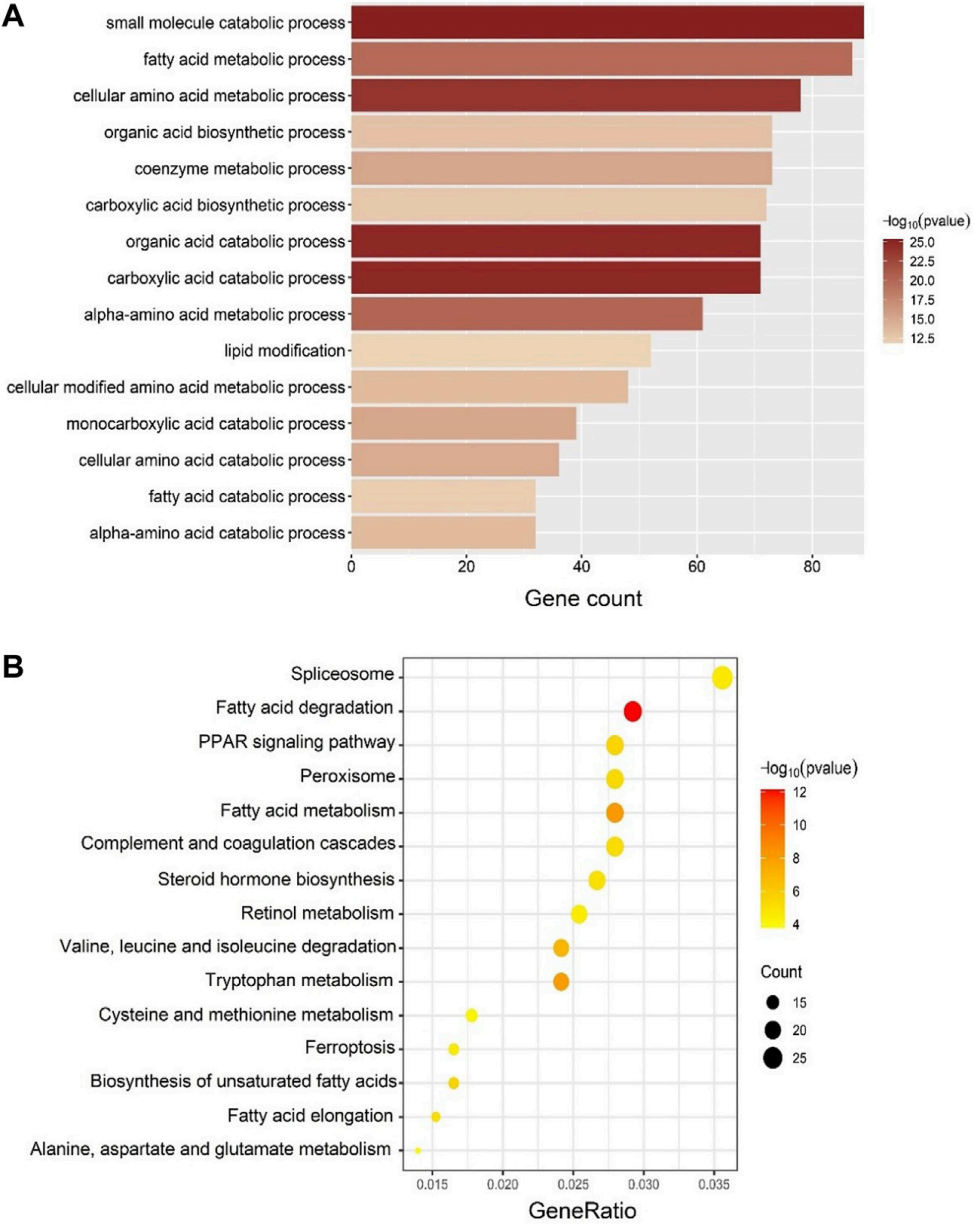
GO and KEGG enrichment analyses in the blue module. **(A)** Top 15 significantly enriched GO biological process (BP) terms. The depth of color corresponds to the enrichment significance of each term, while the *x*-axis represents the gene counts. **(B)** Top 15 significantly enriched KEGG terms. The depth of color corresponds to the enrichment significant of each term, while the size of the dots correlates with the enriched gene counts.

The GO and KEGG enrichment analyses were performed to confirm the biological themes of genes in the blue module and obtain further insights into the underlying biological pathways behind IUGR-hypoxia. The top 15 significant terms of GO and KEGG are exhibited in Figures 5A,B, respectively.

Enriched GO-biological process (BP) terms for the top IUGR-hypoxia module were mainly about metabolic process (Figure 5A), such as “fatty acid metabolic process” (gene count = 87, *p =* 1.24E-20), “lipid modification” (gene count = 52, *p =* 1.44E-12), and “fatty acid catabolic process” (gene count = 32, *p =* 6.57E-13).

The KEGG enrichment results, mainly on lipid metabolism, were similar to those obtained from GO-BP (Figure 5B). The top KEGG terms included “fatty acid degradation” (gene count = 23, *p* = 7.20E-13), “fatty acid metabolism” (gene count = 22, *p* = 6.61E-09), “PPAR signaling pathway” (gene count = 22, *p* = 2.46E-06), and “biosynthesis of unsaturated fatty acids” (gene count = 13, *p* = 2.62E-06).

### 3.6 Identification of Hub Genes

**FIGURE 6:**
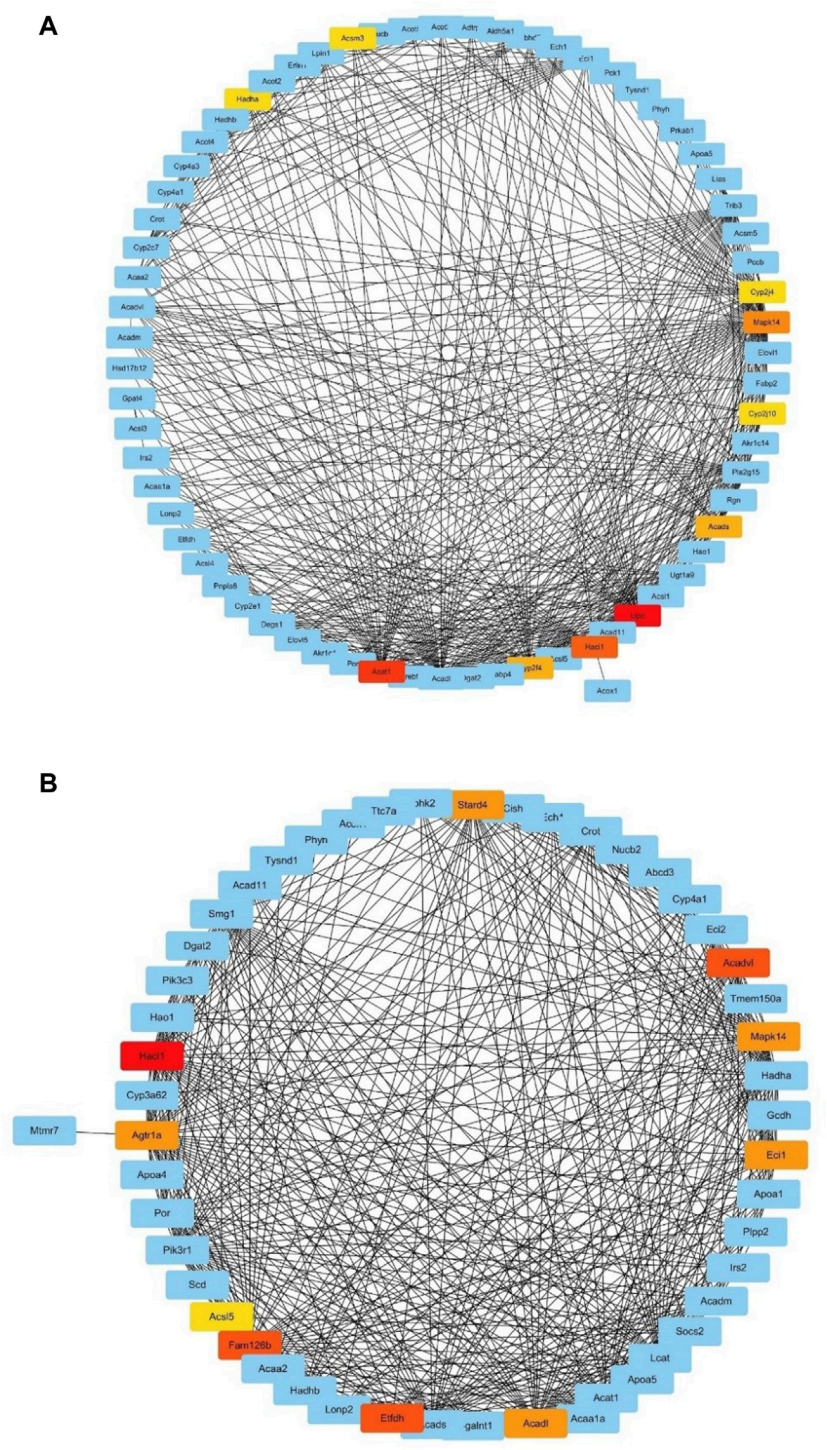
Subnetworks based on maximal clique centrality values. **(A)** Subnetwork of the GO term “fatty acid metabolic process.” **(B)** Subnetwork of the GO term “lipid modification.” The red nodes (higher MCC value) and yellow nodes (lower MCC value) represent genes with the top 10 MCC values.

We extracted the genes enriched in two of the top 15 significant GO-BP terms in the top IUGR-hypoxia module, namely, “fatty acid metabolic process” and “lipid modification” and used them to construct two subnetworks of the weighted co-expression network, respectively. The obtained MCC value of genes predicted by the CytoHubba plugin was used to search for hub genes, and the most central genes of the subnetwork were screened out, as shown in Figure 6. The top 10 genes obtained using the MCC values in the pathway of the fatty acid metabolic process were Hadha, Acsm3, Cyp2j4, Mapk14, Cyp2j10, Acads, Lipc, Hacl1, Cyp2f4, and Acat1, while the top 10 genes in the pathway of lipid modification were Stard4, Acadvl, Mapk14, Ecil, Acadl, Etfdh, Fam126b, Acsl5, Agtrla, and Hacl1.

### 3.7 Validation of Hub Genes Using qRT-PCR

**FIGURE 7:**
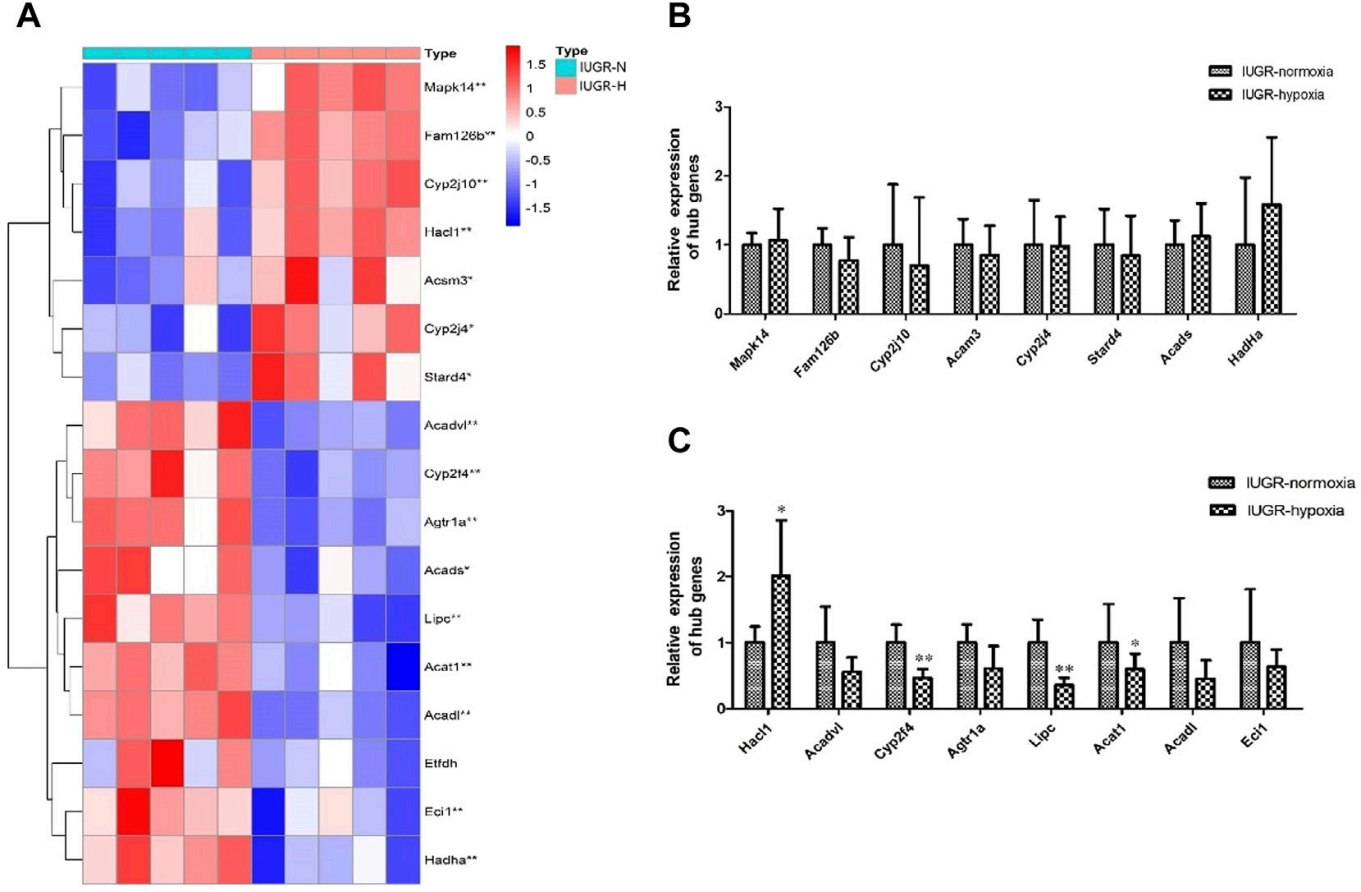
Validation of the hub genes using qRT-PCR. **(A)** Expression of hub genes between the IUGR-normoxia and IUGR-hypoxia groups. **(B)** and **(C)** Validation of the hub genes using qRT-PCR. ^*^ *p* < 0.05, IUGR-normoxia vs. IUGR-hypoxia. ^**^ *p* < 0.01, IUGR-normoxia vs. IUGR-hypoxia.

The expression of hub genes between IUGR-hypoxia and IUGR-normoxia are summarized in Figure 7A. The downregulated hub genes in IUGR-hypoxia included Cyp2f4, Agtr1a, Eci1, Acsl5, Acat1, Acadl, Lipc, Hadha, Acads, and Acadvl, while the upregulated hub genes in IUGR-hypoxia included Acsm3, Cyp2j4, Mapk14, Cyp2j10, Stard4, Fam126b, and Hacl1. The qRT-PCR analysis was conducted in IUGR-normoxia and IUGR-hypoxia liver tissues in order to determine the aberrant expression of IUGR-hypoxia-related hub genes. The obtained results indicated that the mRNA expression of Cyp2f4, Lipc, and Acadl was significantly decreased in IUGR-hypoxia liver tissues when compared with IUGR-normoxia liver tissues, whereas Hacl1 mRNA expression was increased in IUGR-hypoxia liver tissues (Figures 7B,C). These results were in concordance with the WGCNA data.

## Discussion

Accumulating evidence has indicated that PAH is associated with metabolic dysfunction. Previous studies have reported that lipids were abnormally metabolized in PAH patients with reduced HDL (Heresi et al., 2010), elevated plasma FFAs (Brittain et al., 2016), ectopic lipid deposition in the skeletal muscle (Talati et al., 2016), and a possible cardiac lipotoxic phenotype (Hemnes et al., 2014). Pugh et al. (2019) reported that as many as two-thirds of non-diabetic patients with PAH demonstrated some degree of glucose intolerance, defined as a hemoglobin A1c ≥ 6.0%. Further analysis indicated that 15% of the evaluated patients had been newly diagnosed with diabetic mellitus (Pugh et al., 2011). Additionally, PAH was linked with a reduced mitochondrial function characterized by decreased fatty acid oxidation and increased glycolysis in rodent models and cell cultures (Fessel et al., 2012; Archer et al., 2013; Graham et al., 2015). Despite this recognition, little is known about the detailed metabolic pathways and genes influenced by PAH in liver tissue. This study used high-throughput sequencing and WGCNA methods to explore the biological processes and hub genes underlying PAH from a cell’s transcriptional landscape. Our results provided important evidence that the dysfunction of fatty acid metabolism was one of the main problems in animal models of hypoxia PAH following IUGR, which is consistent with changes in hearts and lungs of PAH (Xu et al., 2021). As recently reviewed, fatty acid metabolism would be reprogrammed under hypoxia conditions in the largest metabolic organ of liver through hypoxia-inducible factors (Liu et al., 2014; Bouthelier and Aragonés 2020). On another note, metabolic changes involving fatty acid oxidation is thought to be involved in the formation of PAH (Brittain et al., 2016). Thus, the fatty acid metabolism and PAH interact with each other, serving as both cause and effect.

Next, for further identifying hub genes, the CytoHubba Cytoscape plugin was performed in our study, leading to the identification of 17 hub genes. Moreover, the expression levels of hub genes between the IUGR-control and IUGR-hypoxia groups were validated using RT-qPCR. Four genes were identified: Hacl1, Lipc, Acadl, and Cyp2f4, which are consistent with those obtained by high-throughput sequencing. Hacl1, known as the first peroxisomal enzyme in mammals which has been found to be dependent on TPP (thiamin pyrophosphate), plays an important role in at least two pathways in lipid metabolism: 1) the degradation of 3-methyl-branched fatty acids like phytanic acid and 2) the shortening of 2-hydroxy long-chain fatty acids (Casteels et al., 2007). A previous study reported that mouse models lacking Hacl1 displayed a significant decreased weight, enlarged liver, reduced hepatic triglycerides and glycogen, and absence of abdominal white adipose tissue after dietary administration of phytol (Mezzar et al., 2017). Lipc has been documented for many years as playing the role of a multifunctional protein that acts on metabolism. One study reported a dual function for the Lipc gene which acts as triacylglycerol (TAG) hydrolase and phospholipase and also acts as a ligand (independent of its catalytic activity) for cell surface anchorage/uptake of various lipoproteins (Santamarina-Fojo et al., 2004). In addition, Breckenridge et al. (1982) reported that Lipc deficiency in humans was characterized by an elevation in the plasma concentrations of cholesterol and TAG as well as large and buoyant HDL particles (Breckenridge et al., 1982; Connelly et al., 1990; Hegele et al., 1993). ACADL, a member of the acyl-CoA dehydrogenase superfamily, is an important rate-limiting enzyme relevant to fatty acid oxidation that is responsible for fatty acid activation, translocation, and *β*-oxidation in the mitochondria. Finally, the involvement of Cyp2f4 in metabolism or PAH has not yet been reported. Therefore, further studies are required to fully explore the specific mechanisms of Cyp2f4. All these pathways and genes mentioned in this study might become therapeutic targets with clinical usefulness in the metabolic dysfunction of PAH.

To our knowledge, this study was the first study that established a gene co-expression network based on systems biology-based WGCNA to identify and validate the hub genes associated with metabolism in hypoxia PAH following IUGR. Certainly, this study had several limitations. First, we mainly focused on data mining and data analysis. Second, the analysis accuracy may be influenced because the sample size in our research is relatively small. Finally, basic functional studies of the module and key genes identified here are lacking. Therefore, further experiments are required to better confirm our findings.

## Data Availability

The datasets presented in this study can be found in online repositories. The names of the repository and accession number can be found at: National Center for Biotechnology Information (NCBI) BioProject, https://www.ncbi.nlm.nih.gov/bioproject/, PRJNA779804.

## References

[B1] Archer S. L. Fang Y. H. Ryan J. J. Piao L. (2013). Metabolism and Bioenergetics in the Right Ventricle and Pulmonary Vasculature in Pulmonary Hypertension. Pulm. Circ. 3, 144–152. 10.4103/2045-8932.109960 23662191 PMC3641722

[B2] Assad T. R. Hemnes A. R. (2015). Metabolic Dysfunction in Pulmonary Arterial Hypertension. Curr. Hypertens. Rep. 17, 20. 10.1007/s11906-014-0524-y 25754317 PMC4840894

[B3] Benjamini Y. Hochberg Y. (1995). Controlling the False Discovery Rate: a Practical and Powerful Approach to Multiple Testing. J. R. Stat. Soc. Ser. B (Methodological) 57, 289–300. 10.1111/j.2517-6161.1995.tb02031.x

[B4] Bouthelier A. Aragonés J. (2020). Role of the HIF Oxygen Sensing Pathway in Cell Defense and Proliferation through the Control of Amino Acid Metabolism. Biochim. Biophys. Acta (Bba) - Mol. Cel Res. 1867, 118733. 10.1016/j.bbamcr.2020.118733 32416106

[B5] Breckenridge W. Little J. Alaupovic P. Wang C. Kuksis A. Kakis G. (1982). Lipoprotein Abnormalities Associated with a Familial Deficiency of Hepatic Lipase. Atherosclerosis 45, 161–179. 10.1016/0021-9150(82)90136-8 6961921

[B6] Brittain E. L. Talati M. Fessel J. P. Zhu H. Penner N. Calcutt M. W. (2016). Fatty Acid Metabolic Defects and Right Ventricular Lipotoxicity in Human Pulmonary Arterial Hypertension. Circulation 133, 1936–1944. 10.1161/CIRCULATIONAHA.115.019351 27006481 PMC4870107

[B7] Casteels M. Sniekers M. Fraccascia P. Mannaerts G. P. Van Veldhoven P. P. (2007). The Role of 2-Hydroxyacyl-CoA Lyase, a Thiamin Pyrophosphate-dependent Enzyme, in the Peroxisomal Metabolism of 3-Methyl-Branched Fatty Acids and 2-hydroxy Straight-Chain Fatty Acids. Biochem. Soc. Trans. 35 (Pt 5), 876–880. 10.1042/BST0350876 17956236

[B8] Chen M. Yan J. Han Q. Luo J. Zhang Q. (2020). Identification of Hub‐methylated Differentially Expressed Genes in Patients with Gestational Diabetes Mellitus by Multi‐omic WGCNA Basing Epigenome‐wide and Transcriptome‐wide Profiling. J. Cel Biochem 121, 3173–3184. 10.1002/jcb.29584 31886571

[B9] Chin C.-H. Chen S.-H. Wu H.-H. Ho C.-W. Ko M.-T. Lin C.-Y. (2014). CytoHubba: Identifying Hub Objects and Sub-networks from Complex Interactome. BMC Syst. Biol. 8 (Suppl. 4), S11. 10.1186/1752-0509-8-S4-S11 25521941 PMC4290687

[B10] Connelly P. W. Maguire G. F. Lee M. Little J. A. (1990). Plasma Lipoproteins in Familial Hepatic Lipase Deficiency. Arteriosclerosis 10, 40–48. 10.1161/01.atv.10.1.40 2297346

[B11] Fessel J. P. Hamid R. Wittmann B. M. Robinson L. J. Blackwell T. Tada Y. (2012). Metabolomic Analysis of Bone Morphogenetic Protein Receptor Type 2 Mutations in Human Pulmonary Endothelium Reveals Widespread Metabolic Reprogramming. Pulm. Circ. 2, 201–213. 10.4103/2045-8932.97606 22837861 PMC3401874

[B12] Graham B. B. Kumar R. Mickael C. Sanders L. Gebreab L. Huber K. M. (2015). Severe Pulmonary Hypertension Is Associated with Altered Right Ventricle Metabolic Substrate Uptake. Am. J. Physiology-Lung Cell Mol. Physiol. 309, L435–L440. 10.1152/ajplung.00169.2015 PMC4556932 26115672

[B13] Hegele R. A. Little J. A. Vezina C. Maguire G. F. Tu L. Wolever T. S. (1993). Hepatic Lipase Deficiency. Clinical, Biochemical, and Molecular Genetic Characteristics. Arterioscler Thromb. 13, 720–728. 10.1161/01.atv.13.5.720 8485124

[B14] Hemnes A. R. Brittain E. L. Trammell A. W. Fessel J. P. Austin E. D. Penner N. (2014). Evidence for Right Ventricular Lipotoxicity in Heritable Pulmonary Arterial Hypertension. Am. J. Respir. Crit. Care Med. 189, 325–334. 10.1164/rccm.201306-1086OC 24274756 PMC3977729

[B15] Heresi G. A. Aytekin M. Newman J. DiDonato J. Dweik R. A. (2010). Plasma Levels of High-Density Lipoprotein Cholesterol and Outcomes in Pulmonary Arterial Hypertension. Am. J. Respir. Crit. Care Med. 182, 661–668. 10.1164/rccm.201001-0007OC 20448092 PMC2937236

[B16] Langfelder P. Horvath S. (2008). WGCNA: an R Package for Weighted Correlation Network Analysis. BMC Bioinformatics 9, 559. 10.1186/1471-2105-9-559 19114008 PMC2631488

[B17] Liu Y. Ma Z. Zhao C. Wang Y. Wu G. Xiao J. (2014). HIF-1α and HIF-2α Are Critically Involved in Hypoxia-Induced Lipid Accumulation in Hepatocytes through Reducing PGC-1α-Mediated Fatty Acid β-oxidation. Toxicol. Lett. 226, 117–123. 10.1016/j.toxlet.2014.01.033 24503013

[B18] Luo J. Li H. Liu Z. Li C. Wang R. Fang J. (2020). Integrative Analyses of Gene Expression Profile Reveal Potential Crucial Roles of Mitotic Cell Cycle and Microtubule Cytoskeleton in Pulmonary Artery Hypertension. BMC Med. Genomics 13, 86. 10.1186/s12920-020-00740-x 32586319 PMC7318763

[B19] Mezzar S. De Schryver E. Asselberghs S. Meyhi E. Morvay P. L. Baes M. (2017). Phytol-induced Pathology in 2-Hydroxyacyl-CoA Lyase (HACL1) Deficient Mice. Evidence for a Second Non-HACL1-related Lyase. Biochim. Biophys. Acta (Bba) - Mol. Cel Biol. Lipids 1862, 972–990. 10.1016/j.bbalip.2017.06.004 28629946

[B20] Mura M. Anraku M. Yun Z. McRae K. Liu M. Waddell T. K. (2012). Gene Expression Profiling in the Lungs of Patients with Pulmonary Hypertension Associated with Pulmonary Fibrosis. Chest 141, 661–673. 10.1378/chest.11-0449 21835902

[B21] Napoli C. Benincasa G. Loscalzo J. (2019). Epigenetic Inheritance Underlying Pulmonary Arterial Hypertension. Atvb 39, 653–664. 10.1161/ATVBAHA.118.312262 PMC6436974 30727752

[B22] Pugh M. E. Robbins I. M. Rice T. W. West J. Newman J. H. Hemnes A. R. (2011). Unrecognized Glucose Intolerance Is Common in Pulmonary Arterial Hypertension. J. Heart Lung Transplant. 30, 904–911. 10.1016/j.healun.2011.02.016 21493097 PMC3129440

[B23] Rexhaj E. Bloch J. Jayet P.-Y. Rimoldi S. F. Dessen P. Mathieu C. (2011). Fetal Programming of Pulmonary Vascular Dysfunction in Mice: Role of Epigenetic Mechanisms. Am. J. Physiology-Heart Circulatory Physiol. 301, H247–H252. 10.1152/ajpheart.01309.2010 21536851

[B24] Santamarina-Fojo S. Gonza lez-Navarro H. Freeman L. Wagner E. Nong Z. (2004). Hepatic Lipase, Lipoprotein Metabolism, and Atherogenesis. Atvb 24, 1750–1754. 10.1161/01.ATV.0000140818.00570.2d 15284087

[B25] Schermuly R. T. Ghofrani H. A. Wilkins M. R. Grimminger F. (2011). Mechanisms of Disease: Pulmonary Arterial Hypertension. Nat. Rev. Cardiol. 8, 443–455. 10.1038/nrcardio.2011.87 21691314 PMC7097518

[B26] Shannon P. Markiel A. Ozier O. Baliga N. S. Wang J. T. Ramage D. (2003). Cytoscape: a Software Environment for Integrated Models of Biomolecular Interaction Networks. Genome Res. 13, 2498–2504. 10.1101/gr.1239303 14597658 PMC403769

[B27] Talati M. H. Brittain E. L. Fessel J. P. Penner N. Atkinson J. Funke M. (2016). Mechanisms of Lipid Accumulation in the Bone Morphogenetic Protein Receptor Type 2 Mutant Right Ventricle. Am. J. Respir. Crit. Care Med. 194, 719–728. 10.1164/rccm.201507-1444OC 27077479 PMC5027228

[B28] Wang Y. Chen L. Ju L. Qian K. Liu X. Wang X. (2019). Novel Biomarkers Associated with Progression and Prognosis of Bladder Cancer Identified by Co-expression Analysis. Front. Oncol. 9, 1030. 10.3389/fonc.2019.01030 31681575 PMC6799077

[B29] Xu W. Janocha A. J. Erzurum S. C. (2021). Metabolism in Pulmonary Hypertension. Annu. Rev. Physiol. 83, 551–576. 10.1146/annurev-physiol-031620-123956 33566674 PMC8597719

[B30] Xu X.-F. Lv Y. Gu W.-Z. Tang L.-L. Wei J.-K. Zhang L.-Y. (2013). Epigenetics of Hypoxic Pulmonary Arterial Hypertension Following Intrauterine Growth Retardation Rat: Epigenetics in PAH Following IUGR. Respir. Res. 14, 20. 10.1186/1465-9921-14-20 23406533 PMC3577465

[B31] Yang H. Li H. (2019). CD36 Identified by Weighted Gene Co-expression Network Analysis as a Hub Candidate Gene in Lupus Nephritis. PeerJ 7 (7), e7722. 10.7717/peerj.7722 31592160 PMC6777479

[B32] Zamanian R. T. Hansmann G. Snook S. Lilienfeld D. Rappaport K. M. Reaven G. M. (2009). Insulin Resistance in Pulmonary Arterial Hypertension. Eur. Respir. J. 33, 318–324. 10.1183/09031936.00000508 19047320 PMC2785883

[B33] Zhang Z. Luo X. Lv Y. Yan L. Xu S. Wang Y. (2019). Intrauterine Growth Restriction Programs Intergenerational Transmission of Pulmonary Arterial Hypertension and Endothelial Dysfunction via Sperm Epigenetic Modifications. Hypertension 74, 1160–1171. 10.1161/HYPERTENSIONAHA.119.13634 31596625

