# Supplementary material for: Identification of the Key Pathways and Genes in Hypoxia Pulmonary Arterial Hypertension Following Intrauterine Growth Retardation
Source: Front Mol Biosci. 2022 Mar 31;9:789736. doi: 10.3389/fmolb.2022.789736 (PMC9008831; doi:10.3389/fmolb.2022.789736)
Supplement: Supplementary file 1 [file Table1.DOCX]

**Table 1: The primer sequences of the hub genes.**

| Gene | Sequence | Product |
| --- | --- | --- |
| mitogen-activated  protein kinase 14 (MAPK14) | Forward | GCTGGCTCGGCACACTGATG |
|  | Reverse | GCCCACGGACCAAATATCCACTG |
| family with sequence similarity 126 member B (Fam126b) | Forward | GAGCCTGTCTGCCACCAACTG |
|  | Reverse | GCCATTGCTCTGCCTGTCTCTAC |
| cytochrome P450, family 2, subfamily j, polypeptide 10 (Cyp2j10) | Forward | CGCTGCTGTCACCTTCCTGTTC |
|  | Reverse | TGGCTGCTTCACATCCAACTGG |
| 2-hydroxyacyl-CoA lyase 1 (Hacl1) | Forward | CATGTTCGGTGTCGTAGGCATCC |
|  | Reverse | GCCGCTTGCTCATTCCTCATCC |
| acyl-CoA synthetase medium-chain family member 3 (Acsm3) | Forward | CTGTCTGTCAACGGAAGGTTCTGG |
|  | Reverse | AAACACATGCTCCTTGGGTCCAC |
| cytochrome P450, family 2, subfamily j, polypeptide 4 (Cyp2j4) | Forward | AGAGCTTGCCTTGGAGAACAACTG |
|  | Reverse | GCGGTGCGTGACTGGAGAAAG |
| cytosolic StAR- related lipid transfer domain 4 (STARD4) | Forward | CCTGCGGCTGGTTCTGTGTTC |
|  | Reverse | TGCTTGCCATTGCTGTGTCTACC |
| acyl-CoA synthetase   long-chain family member 5 (ACSL5) | Forward | GGCATCATTCGGCGGAACAG |
|  | Reverse | TGCAGCCCTGAAGAACGTCA |
| acyl-CoA dehydrogenase, very long chain (ACADVL) | Forward | TGTGCTAGGAGAAGTGGGAGATGG |
|  | Reverse | TCAACCGCCTTGGCAATGATGG |
| angiotensin type 1a receptors (AGTR1a) | Forward | GCTTCAACCTCTACGCCAGTGTG |
|  | Reverse | CGAGACTTCATTGGGTGGACGATG |
| cytochrome P450, family 2, subfamily f, polypeptide 4 (Cyp2f4) | Forward | TGTCATCTTCGGCAGTCGTTTCG |
|  | Reverse | CCAGGCACCCAGTCCAGGAG |
| acyl-Coenzyme A dehydrogenase (Acads) | Forward | CTCACAGCAGAAGCAGCAGTGG |
|  | Reverse | TGCCGTTGAGGACCCAGGAG |
| Hepatic lipase  (Lipc) | Forward | AGGTGGCTGCTCTTCTCCTATGG |
|  | Reverse | GCTCCCAGGCTGTACCCAATTAAG |
| acetyl-coA  acetyltransferase 1   (ACAT1) | Forward | CAGACGTGGTGGTGAAGGAAGATG |
|  | Reverse | ATCGTTCAGTGTGCTGGCGTTAG |
| long chain acyl CoA dehydrogenase (Acadl) | Forward | CCCTGGTTTCAGCCTCCATTCAG |
|  | Reverse | CACTTGCCCGCCGTCATCTG |
| hydroxyacyl-CoA dehydrogenase trifunctional multienzyme complex subunit alpha (HADHA) | Forward | GGTGTCTTGCTCCCATGATGTCAG |
|  | Reverse | GAAGCCGAAGCCTGTGGTCAAG |
| Enoyl-CoA  Delta Isomerase 1 (ECI1) | Forward | CCGAGCGTGCCCTTCAACTG |
|  | Reverse | GCCATCACTGAGCGAGCCTTG |
